# The shared KEGG pathways between icariin-targeted genes and osteoporosis

**DOI:** 10.18632/aging.103133

**Published:** 2020-05-07

**Authors:** Tao Yu, Yuan Xiong, Simon Luu, Xiaomeng You, Bing Li, Jiang Xia, Hui Zhu, Youguang Zhao, Haichao Zhou, Guangrong Yu, Yunfeng Yang

**Affiliations:** 1 Department of Orthopedic Surgery, Tongji Hospital, Tongji University School of Medicine, Shanghai 200065, China; 2 Department of Orthopedics, Union Hospital, Tongji Medical College, Huazhong University of Science and Technology, Wuhan 430022, Hubei, China; 3 College of Dental Medicine, Columbia University Irving Medical Center, New York, NY 10032, USA; 4 Department of Orthopedic Surgery, Brigham and Women's Hospital, Harvard Medical School, Boston, MA 02115, USA

**Keywords:** icariin, osteoporosis, bioinformatics, osteogenesis, JNK

## Abstract

Osteoporosis is a common metabolic bone disease that affects about 40% of postmenopausal women. Treatment options for osteoporosis are limited, however. Icariin is an herbal substance that has been shown to improve bone mass, but the mechanisms are largely unknown. Using bioinformatics analysis, we have identified the hub genes and KEGG pathways shared between icariin-targeted genes and osteoporosis. The top five shared KEGG pathways were the Toll-like receptor signaling pathway, adipocytokine pathway, neurotrophin signaling pathway, NOD-like receptor signaling, and B cell receptor signaling pathway; the hub genes were RELA, NFKBIA, and IKBKB, belonging to the NF-κB family. The identified icariin-targeted genes are involved in inflammation, insulin resistance, apoptosis, and immune responses, and regulate the PI3K-Akt, NF-κB, MAPK, and JNK signaling pathways. Our *in vitro* data show that icariin inhibits apoptosis in human mesenchymal stem cells by suppressing JNK/c-Jun signaling pathway. Together, these findings indicate that icariin exerts its anti-osteoporotic function by inhibiting JNK/c-Jun signaling pathway, and suggest that icariin may be a promising treatment option for osteoporosis.

## INTRODUCTION

Osteoporosis is a metabolic bone disease that leads to an increased risk of bone fractures. Patients with osteoporosis have an impaired bone microarchitecture causing decreased bone mass density and increased bone fragility. Osteoporosis has become one of the major problems of public health [1]. Osteoporosis results in 1.5 million fractures per year in the United States [2]. Hip fractures and vertebral fractures are two common types of osteoporotic fractures. Vertebral fractures can cause long-term pain and severely affect the quality of life. Hip fractures make patients unable to stand or walk, and increase the risk of death [1].

Icariin is a natural substance extracted from the herb Epimedium. Icariin has anti-osteoporotic and anti-osteoarthritic effects [3–7], through promoting osteoblastogenesis, and suppressing osteoclastogenesis [8]. However, the mechanisms underlying its anti-osteoporotic action are not fully understood.

Bioinformatics has the potential to identify genes differentially expressed in patients with osteoporosis, predict hub genes based on protein interactions and gene enrichment analyses, and identify the responsible molecular mechanisms [9, 10]. Bioinformatics analysis has been widely used to identify disease mechanisms and search for target genes [11, 12].

In this study, we used bioinformatics analysis to identify the icariin-targeted genes and the signaling pathways involved in osteoporosis. Using gene enrichment analysis, shared Kyoto Encyclopedia of Genes and Genomes (KEGG) pathways of icariin-targeted genes and osteoporosis were identified, and the hub genes were obtained. Using simulated drawing, our data indicate that icariin exerts its biological effects through regulating the PI3K-Akt, NF-kB, MAPK, and JNK signaling pathways. Our *in vitro* data show that icariin inhibits apoptosis of human mesenchymal stem cells by suppressing the JNK signaling pathway. Together, our study suggests that icariin may be a promising treatment option for osteoporosis.

## RESULTS

### Icariin-targeted genes and interaction networks

**Figure 1:**
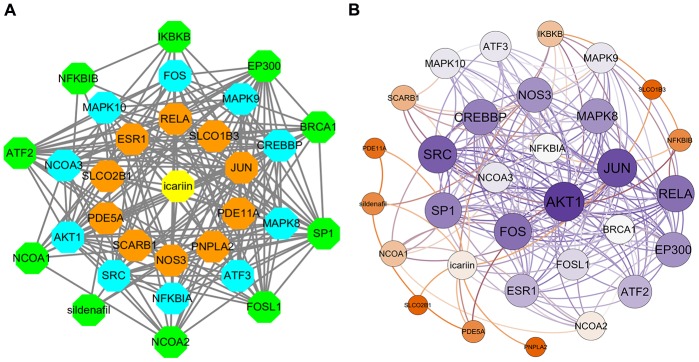
**The interaction networks of icariin-targeted genes.** (**A**) Interaction network constructed by Cytoscape. (**B**) Weighted interaction network.

A total of 29 icariin-interacting genes were identified using the Search Tool for Interacting Chemicals (STITCH) database. STITCH is a database that integrates various chemicals into a single resource. Icariin-targeted genes were obtained in STITCH using the default settings. The first shell (chemical-protein) was RELA, JUN, PDE5A, NOS3, ESR1, PDE11A, SCARB1, PNPLA2, SLCO2B1, and SLCO1B3. The second shell (protein-protein) was CREBBP, NCOA3, MAPK9, SRC, MAPK8, AKT1, ATF3, MAPK10, FOS, and NFKBIA. The third shell (protein-protein and chemical) was IKBKB, NCOA1, BRCA1, NCOA2, SP1, EP300, ATF2, FOSL1, NFKBIB, and sildenafil. The interaction of icariin-targeted genes was constructed using Cytoscape 3.7.2 (Figure 1A). Then the visualization of the network based on interaction weights was constructed, indicating that AKT1 and JUN had the highest weight (Figure 1B).

Using the Database for Annotation, Visualization, and Integrated Discovery (DAVID), 71 icariin-related KEGG pathways were obtained; 58 KEGG pathways with *p*-value ≤ 0.01 were selected for further analyses. Using the miRWalk database, 110 osteoporosis-related KEGG pathways were obtained.

### Identification of shared KEGG pathways and enrichment analysis

**Figure 2:**
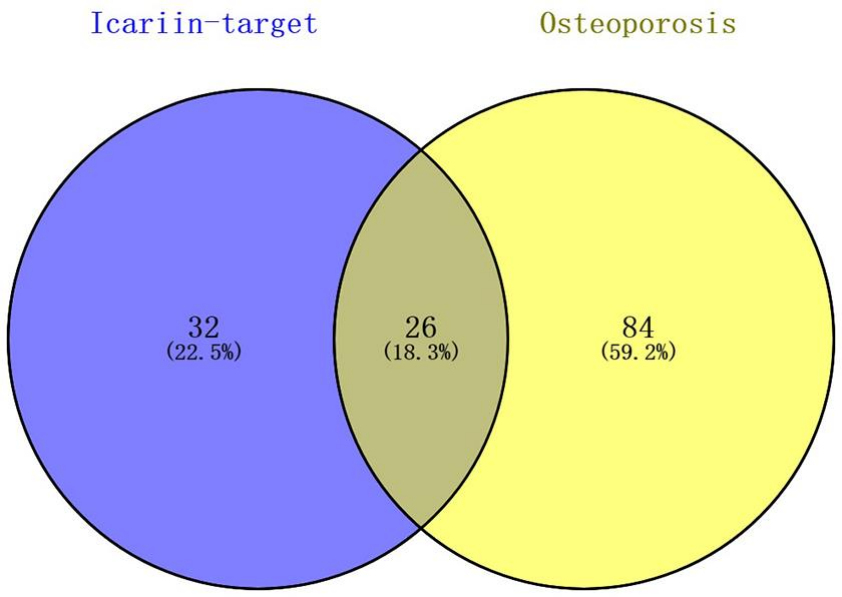
**Identification of shared KEGG pathways of icariin-targeted genes and osteoporosis.** 58 icariin-targeted genes related KEGG pathways, and 110 osteoporosis related KEGG pathways were found; 26 (18.3%) shared KEGG pathways were identified.

**Figure 3:**
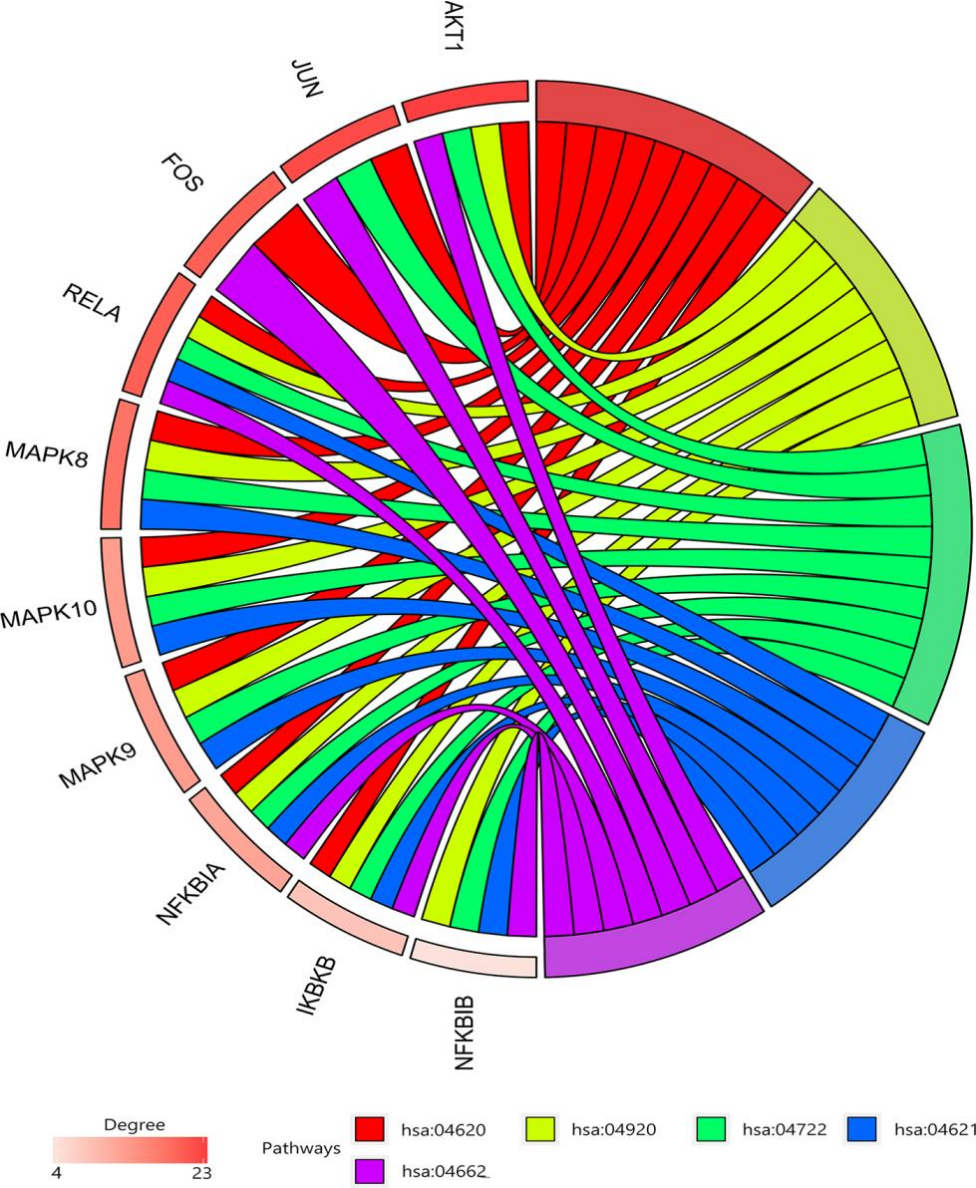
**Gene enrichment analysis.** RELA, NFKBIA, and IKBKB were involved in all five pathways. The top three genes by degree were AKT1, JUN, and SRC.

**Figure 4:**
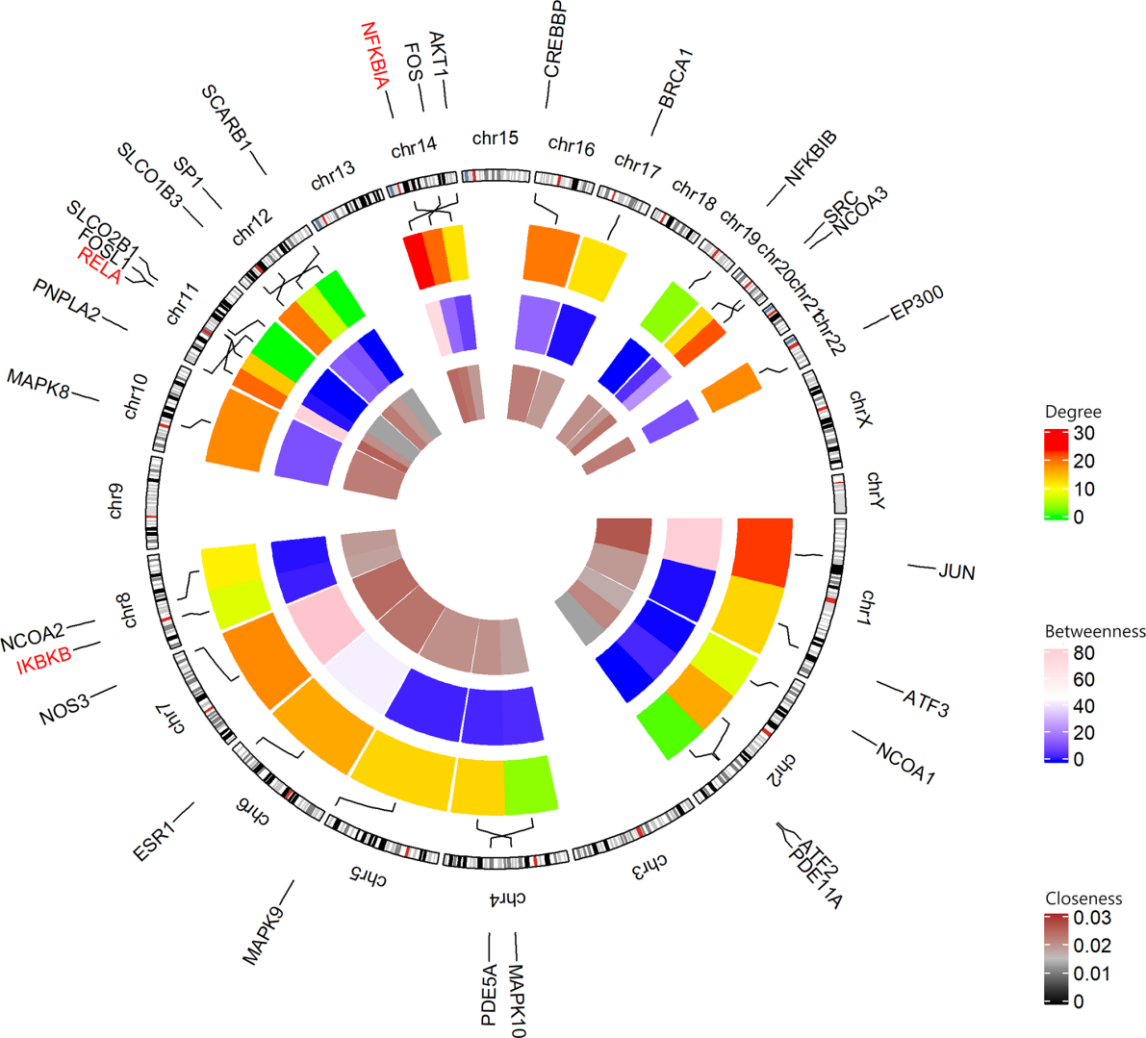
**Circular visualization of chromosomal positions and connectivity of icariin-targeted genes.** The names of the genes are shown in the outer circle. For the outer heatmap, red represents high degree, and green represents low degree. For the middle heatmap, pink represents high betweenness, and blue represents low betweenness. For the inner heatmap, brown represents high closeness, and black represents low closeness. Lines coming from each gene point to their specific chromosomal locations on the chromosomal circle. The three hub genes are shown in red.

26 shared KEGG pathways of icariin-targeted genes and osteoporosis were identified with Venn Diagram (Figure 2). The top five KEGG pathways were the Toll-like receptor signaling pathway, Adipocytokine signaling pathway, Neurotrophin signaling pathway, NOD-like receptor signaling pathway, and B cell receptor signaling pathway (Table 1). Since the NF-κB-related genes RELA, NFKBIA, and IKBKB were involved in all five KEGG pathways, they were considered as the hub genes. The gene enrichment analysis results obtained using the GOplot tool [13], are shown in Figure 3. The information on chromosomal positions and connectivity of icariin-targeted genes was obtained using the circular visualization tool [14]; it is shown in Figure 4. The NF-κB genes RELA, NFKBIA, and IKBKB are located on chr11, chr14, and chr8, respectively.

**Table 1:** Top five KEGG pathway and related genes.

**Term**	**KEGG Pathway**	**Icariin-targeted Genes**	***P*-value**
hsa04620	Toll-like receptor signaling pathway	AKT1, FOS, JUN, RELA, NFKBIA, MAPK9, MAPK8, MAPK10, IKBKB	4.2E-9
hsa04920	Adipocytokine signaling pathway	AKT1, RELA, NFKBIB, NFKBIA, MAPK9, MAPK8, MAPK10, IKBKB	6.3E-9
hsa04722	Neurotrophin signaling pathway	AKT1, JUN, RELA, NFKBIB, NFKBIA, MAPK9, MAPK8, MAPK10, IKBKB	1.1E-8
hsa04621	NOD-like receptor signaling pathway	RELA, NFKBIB, NFKBIA, MAPK9, MAPK8, MAPK10, IKBKB	5.7E-8
Hsa04662	B cell receptor signaling pathway	AKT1, FOS, JUN, RELA, NFKBIB, NFKBIA, IKBKB	2.0E-7

### Retrieval of icariin-related KEGG pathways

**Figure 5:**
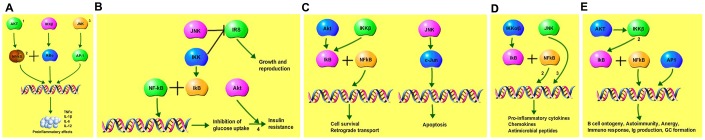
**Icariin-targeted genes related to the top 5 shared KEGG pathways.** (**A**) Icariin-targeted genes related to Toll-like receptor pro-inflammatory signaling: (1) PI3K-Akt signaling, (2) NF-kB, and (3) MAPK signaling pathway. (**B**) Icariin-targeted genes related to Adipocytokine signaling, and insulin resistance. (**C**) Icariin-targeted genes related to Neurotrophin signaling regulating cell survival and apoptosis. (4) JNK signaling pathway is involved in the process. (**D**) Icariin-targeted genes related to NOD-like receptor signaling pathway. (2) NF-kB, and (3) MAPK signaling pro-inflammatory pathways are involved. (**E**) Icariin-targeted genes related to B cell receptor signaling. (2) NF-kB pathway is involved, regulating B cell ontogeny, autoimmunity anergy, and immune responses.

The icariin-targeted genes related to the top 5 shared KEGG pathways are shown in Figure 5. These genes are involved in inflammation, insulin resistance, apoptosis, antimicrobial peptides, and immune responses. They are involved in PI3K-Akt, NF-κB, MAPK, and JNK signaling pathways.

### Icariin inhibits apoptosis of human mesenchymal stem cells (hMSCs)

**Figure 6:**
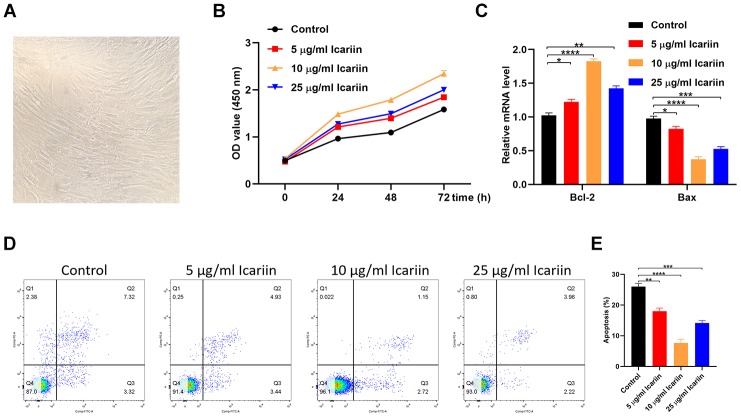
**Icariin inhibits apoptosis of hMSC cells in vitro.** (**A**) The cellular morphology of hMSCs; (**B**) CCK-8 proliferation assay of icariin-treated hMSCs; (**C**) Bcl-2 and Bax gene expression in icariin-treated hMSCs measured by qRT-PCR; (**D**, **E**) Apoptosis assessed by flow cytometry.

To investigate the icariin function, we analyzed its effect on *in vitro* proliferation of hMSC cells using the cell counting kit 8 (CCK-8). As shown in Figure 6A, 6B, treatment of hMSC cells with 10 μg/ml icariin significantly increased their proliferation. In addition, icariin increased expression of the anti-apoptotic gene Bcl-2, while it decreased expression of the pro-apoptotic gene Bax (Figure 6C). The primer sequences are displayed in Table 2. Flow cytometry revealed a significant increase in cells entering the S phase following icariin treatment, with a corresponding decrease in hMSC apoptosis (Figure 6D, 6E). These results indicate that icariin inhibits apoptosis of hMSC cells.

**Table 2:** The primers used in the experiments.

**Gene name**	**Primer sequence**
hsa - Bcl-2 - Forward	GATAACGGAGGCTGGGATGC
hsa - Bcl-2 - Reverse	TCACTTGTGGCCCAGATAGG
hsa - Bax - Forward	CCCTTTTGCTTCAGGGTTTC
hsa - Bax - Reverse	GAGACACTCGCTCAGCTTCTTG
hsa - GAPDH - Forward	CCGTTGAATTTGCCGTGA
hsa - GAPDH - Reverse	TGATGACCCTTTTGGCTCCC

### Icariin inhibits apoptosis of hMSC cells by suppressing c-Jun/JNK signaling

**Figure 7:**
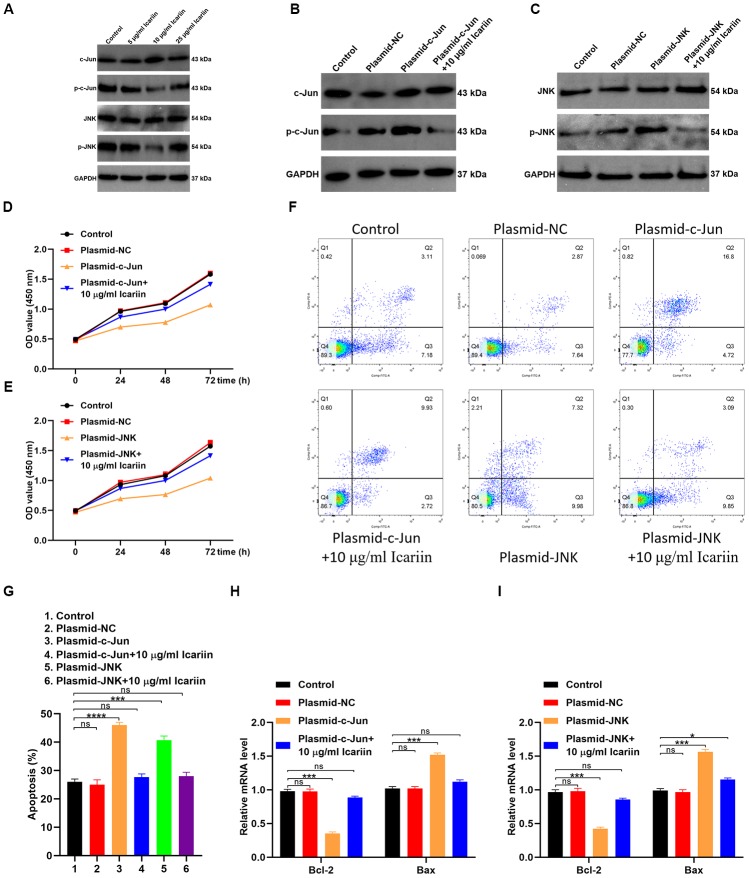
**Icariin inhibits apoptosis of hMSCs by suppressing c-Jun/JNK signaling.** (**A**) Expression of c-Jun, p-c-Jun, JNK, and p-JNK analyzed in icariin-treated hMSCs by western blotting; (**B**, **C**) Phosphorylation levels of c-Jun and JNK in hMSCs transfected with c-Jun or JNK plasmids and treated with icariin (10 μg/ml), analyzed by western blotting; (**D**, **E**) CCK-8 proliferation assay of icariin-treated hMSCs; (**F**) Apoptosis analyzed by flow cytometry of icariin-treated hMSCs; (**G**) Apoptosis analyzed in hMSCs transfected with c-Jun and JNK plasmids, and treated with icariin; (**H**, **I**) Bcl-2 and Bax mRNA levels analyzed by qRT-PCR.

To evaluate the molecular mechanisms by which icariin inhibits apoptosis of hMSC cells, we first analyzed phosphorylated levels of c-Jun (p-c-Jun) and c-Jun N-terminal kinase (p-JNK) by western blotting. As shown in Figure 7A, icariin decreased the phosphorylated levels of p-c-Jun and p-JNK, indicating that it inhibits c-Jun and JNK activation in hMSC cells. Furthermore, icariin (10 μg/ml) decreased the levels of p-c-Jun and p-JNK in cells transfected with c-Jun or JNK plasmids (Figure 7B, 7C). Transfection of hMSC cells with c-Jun or JNK plasmids reduced their proliferation, but icariin (10 μg/ml) partly reversed this effect (Figure 7C, 7D). Flow cytometry revealed a significant recovery in the number of cells entering the S phase following icariin treatment, with a corresponding decrease in hMSC apoptosis (Figure 7F, 7G). In addition, icariin increased gene expression of Bcl-2, but decreased expression of Bax in hMSC cells (Figure 7H, 7I). Together, these results indicate that icariin inhibits apoptosis of hMSC cells by suppressing the c-Jun/JNK signaling pathway.

## DISCUSSION

Osteoporosis is a metabolic bone disease caused by an imbalance in bone turnover, resulting in an increased risk of bone fractures. The treatment of osteoporosis is still a challenge [15]. Icariin is an herbal substance extracted from the plant Epimedium, which has anti-osteoporotic and anti-osteoarthritic effects [3–7], and promotes bone healing [16]. Several studies have suggested that icariin not only increases the osteogenic differentiation of bone mesenchymal stem cells [17–19], but also inhibits osteoclast differentiation [20]. In addition, icariin can improve bone formation [21], and prevent ovariectomy-induced bone loss [22]. Therefore, icariin might improve osteoporosis by promoting osteoblastogenesis and suppressing osteoclastogenesis [8], but the specific mechanisms are not clear.

In this study, we found 26 shared KEGG pathways between icariin-targeted genes and osteoporosis. The top five of the 26 KEGG pathways ranked by increasing p value were the Toll-like receptor signaling pathway, Adipocytokine signaling pathway, Neurotrophin signaling pathway, NOD-like receptor signaling pathway, and B cell receptor signaling pathway. The NF-κB genes RELA, NFKBIA, and IKBKB were identified as the hub genes, because they were involved in all five pathways. The information on chromosomal positions and connectivity of related genes is shown in Figure 4. Degree Centrality represents the association degree of one node and all the other nodes in the network. Closeness Centrality is the close degree of a node and other nodes in the network. Betweenness Centrality is the number of times that a node acts as the shortest bridge between the other two nodes. The hub genes RELA, NFKBIA, and IKBKB appear in all five pathways, indicating that they are involved in their regulation; however, since their degrees are not the highest, they may not have a central role in these pathways.

Using simulated drawing of the icariin-targeted genes related to the five KEGG pathways, our data indicate that icariin exerts its biological effects through regulating the PI3K-Akt, NF-kB, MAPK, and JNK signaling pathways. The identified icariin-targeted genes are associated with inflammation, insulin resistance, apoptosis, antimicrobial peptides, and immune response.

The kinases belonging to the JNK family play a critical role in regulating apoptosis [23]. MAPK8, MAPK9, and MAPK10 are JNKs involved in stimulating apoptotic signaling [23]. c-Jun is a protein encoded by the JUN gene downstream to JNK (Figure 5C). MAPK8, MAPK9, MAPK10, and JUN are all target genes of icariin. In the interaction network of icariin and its target genes, JUN was in the first shell, and MAPK8, MAPK9, MAPK10 were in the second shell (Figure 1A). Since our results showed that icariin inhibits apoptosis of hMSC cells (Figure 6), we hypothesized that it might suppress activities of MAPK8, MAPK9, MAPK10, and JUN kinases. Indeed, our results showed that icariin decreases the phosphorylated levels of JNK and c-Jun in hMSC cells (Figure 7), indicating that it inhibits activation of JNK and c-Jun. The interaction network of icariin and its target genes indicates that icariin inhibits apoptosis by directly inhibiting JUN, or indirectly acting on MAPK8, MAPK9, and MAPK10 (Figure 1).

There are many subtypes of osteoporosis, the most common being postmenopausal osteoporosis. In this study, we have only focused on the anti-apoptotic effects of icariin through regulating the JNK signaling pathway. However, since icariin likely regulates other anti-apoptotic pathways, future studies should systematically analyze the mechanisms responsible for its anti-apoptotic effects. In addition, it will be important to analyze its effect in different subtypes of osteoporosis, and compare its efficacy with other anti-osteoporotic drugs.

In conclusion, our bioinformatics analysis indicates that icariin exerts its anti-osteoporotic functions through regulating the Toll-like receptor, Adipocytokine, Neurotrophin, NOD-like receptor, and B cell receptor signaling pathways. Our *in vitro* data indicate that icariin inhibits apoptosis by suppressing activation of JNK and c-JUN. Even though more studies are needed, these data suggest that icariin may become a promising drug for treating osteoporosis.

## MATERIALS AND METHODS

### Icariin-targeted genes prediction and interaction network construction

The icariin-targeted genes were screened using the STITCH online search tool (http://stitch.embl.de/). STITCH is a web-based tool used to identify and predict interactions among genes, proteins, and chemicals [24]. Following importing “Icariin” into STITCH, the interaction network of icariin and its target genes was calculated using the following calculation settings: three shells with the maximum number of interactions 10 of each shell; medium confidence score 0.4. The interaction network of icariin and its targeted genes was constructed in Cytoscape 3.7.2 [25], and the weighted network was constructed in Gephi.

### Gene enrichment and KEGG pathway analyses

The database for Annotation, Visualization, and Integrated Discovery (DAVID) bioinformatics resources consists of an integrated biological knowledgebase and analytic tools aimed at systematically extracting biological meaning from large gene/protein lists [26, 27]. The icariin-targeted genes were imported into the DAVID database to perform the KEGG pathway and gene enrichment analyses [26, 27].

### Shared KEGG pathways involved in osteoporosis and icariin-targeted genes

MiRWalk, a comprehensive atlas of predicted and validated miRNA-target interactions [28], was used to retrieve the KEGG pathways involved in osteoporosis. The intersection of icariin-targeted genes was associated with the KEGG pathways (*p*≤0.01). Osteoporosis related KEGG pathways were obtained using Venn Diagram (Venny 2.1, http://bioinfogp.cnb.csic.es/tools/venny/index.html), and the top five shared KEGG pathways with smallest p values were selected.

### Identification of the hub genes

The enrichment information of the top five KEGG pathways was presented with GOplot, an R package for visually combining expression data with functional analysis [13]. Genes involved in all five top shared KEGG pathways were considered as hub genes. The information of centrality in the network and position in the chromosomal of all icariin-targeted genes were presented with circlize R package [14].

### Retrieval of KEGG pathways related to icariin-targeted genes

The top 5 shared KEGG pathways with the smallest p values were selected. The KEGG pathways related to icariin-targeted genes were established with the Pathway Builder Tool 20 (https://www.proteinlounge.com).

### Cell culture and transfection

Human mesenchymal stem cells (hMSCs) were obtained from the Orthopedic Laboratory of Tongji Hospital, Tongji University, and cultured in F12 medium (Gibco; Thermo Fisher Scientific, Inc.) with 10% fetal bovine serum (Gibco; Thermo Fisher Scientific, Inc.) and 1% penicillin/streptomycin (Gibco; Thermo Fisher Scientific, Inc.). Lipofectamine® 3000 (Thermo Fisher Scientific, Inc.) was used to transfect cells with plasmid RNA oligos. Plasmid-c-Jun and plasmid-JNK (Guangzhou RiboBio Co., Ltd.) were transfected at a concentration of 50 nM, and 48 hours after transfection, cells were collected for western blotting and qRT-PCR analyses.

### qRT-PCR analysis

TRIzol® (Thermo Fisher Scientific, Inc.) was used to isolate total RNA, and the purified RNA was reverse transcribed into cDNA using the ReverTra Ace® qPCR RT Master Mix (Toyobo Life Science). The RT reaction was conducted for 15 minutes at 42°C, followed by 5 minutes at 98°C; the reaction volume was 20 μl. The qPCR thermocycling conditions were: initial denaturation at 95°C for 30 seconds; 40 cycles at 95°C for 5 seconds, and 60°C for 30 seconds; the reaction volume was 25 μl. GAPDH served as an internal control. Relative mRNA expression was normalized to GAPDH, and calculated according to the 2-ΔΔCq method. All experiments were conducted in triplicates.

### Western blotting analysis

Cell lysates were prepared using NETN buffer (20 mM Tris-HCl, pH 8.0, 100 mM NaCl, 1 mM EDTA and 0.5% Nonidet P-40) and resolved by SDS-PAGE. Proteins were transferred to PVDF membranes and blocked in 5 % skimmed milk at 4°C overnight. Membranes were probed with primary antibodies and labeled with HRP-conjugated secondary antibodies (Aspen, Johannesburg, South Africa). Chemiluminescence detection system (Canon, Tokyo, Japan) was used to visualize the protein bands.

### CCK-8 assay

Six replicates of 103 hMSCs were added to 96-well plates and cultured for 24, 48, or 72 hours. Subsequently, CCK-8 reagent was added to cells in serum-free medium for 2 hours, followed by measurements of absorbance at 450 nm.

### Cell apoptosis assay

Cell apoptosis was determined using Annexin V-FITC/PI apoptosis detection kit (eBioscience, USA) and flow cytometry.

### Statistical analysis

GraphPad Prism 8.0 (GraphPad Software, Inc.) was used to conduct all analyses; the data are presented as the mean ± standard deviation. The Student’s t-test was used to compare two groups of data, whereas groups of 3 or more were compared using one-way analysis of variance with Tukey’s post-hoc test. P<0.05 was considered to be statistically significant. All experiments were performed three times.
